# Body macronutrient composition is predicted by lipid and not protein content of the diet

**DOI:** 10.1002/ece3.3529

**Published:** 2017-10-22

**Authors:** Joshua P. Moatt, Catherine Hambly, Elizabeth Heap, Anna Kramer, Fiona Moon, John R. Speakman, Craig A. Walling

**Affiliations:** ^1^ School of Biological Sciences Institute of Evolutionary Biology University of Edinburgh Edinburgh UK; ^2^ Institute of Biological and Environmental Sciences University of Aberdeen Aberdeen UK; ^3^ Edinburgh Genomics Roslin Institute University of Edinburgh Edinburgh UK; ^4^ State Key Laboratory of Molecular Developmental Biology Institute of Genetics and Developmental Biology Chinese Academy of Sciences Guangzhou Shi China

**Keywords:** body composition, diet, dietary restriction, fat storage, nutrition

## Abstract

Diet is an important determinant of fitness‐related traits including growth, reproduction, and survival. Recent work has suggested that variation in protein:lipid ratio and particularly the amount of protein in the diet is a key nutritional parameter. However, the traits that mediate the link between dietary macronutrient ratio and fitness‐related traits are less well understood. An obvious candidate is body composition, given its well‐known link to health. Here, we investigate the relationship between dietary and body macronutrient composition using a first‐generation laboratory population of a freshwater fish, the three‐spine stickleback (*Gasterosteus aculeatus*). Carbohydrate is relatively unimportant in the diet of predatory fish, facilitating the exploration of how dietary protein‐to‐lipid ratio affects their relative deposition in the body. We find a significant effect of lipid intake, rather than protein, on body protein:lipid ratio. Importantly, this was not a result of absorbing macronutrients in relation to their relative abundance in the diet, as the carcass protein:lipid ratios differed from those of the diets, with ratios usually lower in the body than in the diet. This indicates that individuals can moderate their utilization, or uptake, of ingested macronutrients to reach a target balance within the body. We found no effect of diet on swimming endurance, activity, or testes size. However, there was an effect of weight on testes size, with larger males having larger testes. Our results provide evidence for the adjustment of body protein:lipid ratio away from that of the diet. As dietary lipid intake was the key determinant of body composition, we suggest this occurs via metabolism of excess protein, which conflicts with the predictions of the protein leverage hypothesis. These results could imply that the conversion and excretion of protein is one of the causes of the survival costs associated with high‐protein diets.

## 1 INTRODUCTION

Variation in diet is well known to be a critical determinant of fitness‐related traits such as growth, reproduction, and survival (Fontana & Partridge, 2015; Partridge, Gems, & Withers, 2005). In particular, dietary restriction (DR), a reduction in the intake of calories or particular macronutrients, has been shown to extend lifespan and protect against age‐related diseases in the majority of species studied to date (see Speakman & Mitchell, 2011; Nakagawa, Lagisz, Hector, & Spencer, 2012; Selman, 2014 for recent reviews). It is widely accepted that this lifespan extension can be achieved through a reduction in calorie intake (McCay, Crowell, & Maynard, 1935; reviewed Speakman & Mitchell, 2011). However, recent research has rejuvenated the suggestion that variation in the ratio of specific macronutrients, and in particular a reduction in the protein content of the diet, is a key component of the relationship between diet and lifespan (Carey et al., 2008; Lee et al., 2008; Maklakov et al., 2008; Fanson, Weldon, Pérez‐Staples, Simpson, & Taylor, 2009; Solon‐Biet et al., 2014; Jensen, McClure, Priest, & Hunt, 2015; but see Speakman, Mitchell, & Mazidi, 2016; Simpson et al., 2017 for discussion). Despite this interest, the traits that link dietary macronutrient intake and lifespan are not currently known. An obvious starting point is the relationship between dietary macronutrient ratio and body composition, especially given the importance of body composition and particularly fat deposition, in determining health and lifespan (Barzilai, Banerjee, Hawkins, Chen, & Rossetti, 1998; Muzumdar et al., 2008). Here, using a freshwater fish as our model, we investigate the relationship between macronutrient ratio of the diet and body composition, as well as how macronutrient ratio impacts on physical performance and activity, two indicators of health and lifespan.

Calorie restriction is well known to affect body weight (McCay et al., 1935) but is also suggested to affect body composition, particularly adiposity (Colman, Roecker, Ramsey, & Kemnitz, 1998; Hempenstall, Picchio, Mitchell, Speakman, & Selman, 2010; Mitchell et al., 2015; Muzumdar et al., 2008; Picard & Guarente, 2005) and relative organ size (Mitchell et al., 2015; Selman et al., 2005). In fact, it has been suggested that a reduction in adiposity is the primary mechanism through which calorie restriction acts to extend health and lifespan (Barzilai et al., 1998; Muzumdar et al., 2008; Picard & Guarente, 2005). In mice, for example, adipose loss due to calorie restriction occurs in a graded manner, mirroring that of lifespan extension (Mitchell et al., 2015). However, contradictory evidence suggests that fat loss under calorie restriction provided no benefit or was detrimental to lifespan (Chiba et al., 2014; Liao et al., 2011; Park et al., 2017). Thus, although body composition appears to play a role in mediating the effect of calorie restriction on lifespan, the exact nature of this relationship is currently unclear.

Similar to calorie restriction, changes in dietary macronutrient composition result in changes to both body composition and lifespan. For example, it has been shown that mice fed high protein:carbohydrate ratio diets have reduced body fat (Huang et al., 2013; Solon‐Biet et al., 2014; Sørensen, Mayntz, Raubenheimer, & Simpson, 2008), but surprisingly not the longest lifespan (Solon‐Biet et al., 2014). However, a different study found little to no effect of changing dietary protein:carbohydrate ratio on body fat mass (Mitchell et al., 2015). In *Drosophila melanogaster*, body weight and lipid‐free bodyweight increased with increasing protein:carbohydrate ratio of the diet, with carcass lipid content highest on a dietary protein:carbohydrate ratio of 1:2 (Lee, 2015). These flies had the second highest mean and maximum lifespans, with lifespan maximized on a 1:4 diet. However, additional studies in *D. melanogaster* found that with increasing protein intake, there was a decrease in body weight, due to a decline in body fat (Ponton et al., 2015; Skorupa, Dervisefendic, Zwiener, & Pletcher, 2008). Thus, as with calorie restriction, although dietary macronutrient ratio appears to influence body composition, the relationship between diet and body composition and lifespan appears complex.

Improving our understanding of how variation in dietary macronutrient ratio influences body composition may shed light on the causes of the lifespan cost of being fed imbalanced diets. An obvious candidate is that there are metabolic or storage costs of excess nutrients merely being absorbed in relation to their relative abundance in the diet. It is known that the body has a limited capacity for storing excess protein, with surplus nitrogen being excreted as urea (Delimaris, 2013; Heaney, 1998; Tarnopolsky et al., 1992). However, there is a positive relationship between fat intake and fat storage, with ingestion of high‐fat diets resulting in increased fat storage and obesity and thus potentially the associated negative consequences for health and survival (reviewed Hariri & Thibault, 2010; but see Liao et al., 2011; Chiba et al., 2014; Park et al., 2017). The protein leverage hypothesis suggests that individuals eat primarily to obtain a target protein level, with carbohydrate and fat being overconsumed on low‐protein diets in an attempt to reach this protein level (Huang et al., 2013; Simpson & Raubenheimer, 2005; Sørensen et al., 2008). Although this hypothesis focuses on protein intake, it can be predicted that this modification of food intake in relation to protein availability will also affect body composition (Simpson & Raubenheimer, 2005). For example, when eating to a target protein intake, nonprotein constituents are consumed in relation to their abundance in the diet. Therefore, across multiple diets with varying ratios of protein:nonprotein, we would expect the protein content of the body to remain stable, but the content of other components to vary in relation to their relative abundance. Studies from agriculture and aquaculture would seem to support this; when protein is limiting, individuals appear to prioritize protein ingestion and consequently overconsume lipid and carbohydrate, resulting in greater adiposity (Aletor, Hamid, Niess, & Pfeffer, 2000; Andrews & Ørskov, 1970; Donaldson, Combs, & Romoser, 1956; Ruohonen, Koskela, Vielma, & Kettunen, 2003; Ruohonen, Simpson, & Raubenheimer, 2007). If metabolic or storage costs of excess nutrients are driving the cost of imbalanced diets, we would expect that the protein:lipid ratio of the carcass would be similar to that of the diet and would have the same rank order of protein:lipid ratios as the diets.

An alternative explanation for the survival cost of imbalanced diets is that animals have the potential to selectively absorb and or excrete particular nutrients and that the cost of an imbalanced diet is due to the costs of these selective processes (Fanson, Fanson, & Taylor, 2012). Under this scenario, body and diet macronutrient compositions would not be expected to match, but body compositions would be expected to be more similar than diet compositions, as individuals selectively absorb or excrete particular nutrients in attempt to reach a target protein:lipid ratio within the body. If individuals are targeting a specific carcass protein:lipid ratio, then the protein content of the carcass would differ across diets. Furthermore, we would expect to see clustering and a reduction in variability in carcass protein:lipid ratio, as individuals would be trying to achieve a particular protein content in relation to their lipid content.

In addition to body composition, physical activity and performance (e.g., endurance) are commonly linked with health and lifespan and are affected by diet. It has been suggested that an increase in activity in response to short‐term food shortage would improve an individual's ability to find new food sources, thus explaining the commonly observed biphasic pattern of activity (reviewed Speakman & Mitchell, 2011). However, recent evidence suggests that the effect of calorie restriction differs between different components of activity (Mitchell et al., 2016). Currently, there is little to no exploration of how shortage of a specific macronutrients, rather than overall calorie deficit, affects activity and endurance.

Finally, the effect of diet appears to be sexually dimorphic, with lifespan extension under DR greater in females than males (Nakagawa et al., 2012 but see Speakman et al., 2016). It is thought that this sex difference is a result of a differences between males and females in their investment in reproduction (Shanley & Kirkwood, 2000; but see Moatt, Nakagawa, Lagisz, & Walling, 2016), but work exploring the effect of DR on reproduction in males is often lacking (Moatt et al., 2016). One measure of reproductive investment in males is testes mass, but this is often difficult to study as it would require sacrificing males in studies where lifespan is the key trait of interest. In mice, it has been shown that testes mass is only reduced at high restriction levels, suggesting testes are protected against the effect of DR (Mitchell et al., 2015). The same study reported a marginal effect of protein restriction on testes mass (Mitchell et al., 2015), with a further study reporting increased testes mass on high protein:carbohydrate ratios (Solon‐Biet et al., 2015). However, very few other studies look at the effect of dietary macronutrients on testes mass.

Here, we used three‐spined sticklebacks (*Gasterosteus aculeatus*) reared on diets that varied in macronutrient ratio to investigate the following questions: (1) what is the effect of macronutrient intake on growth and body composition and is this driven by variation in protein content of the diet; (2) how does macronutrient manipulation affect activity and swimming endurance; (3) are there sex differences in the effect of macronutrient manipulation; and (4) what is the effect of macronutrient manipulation on testes size? We predicted that growth would be highest on the diet with the best balance, containing high levels of both protein and lipid. In line with the protein leverage hypothesis, we expect the rank order of carcass protein:fat ratios will match that of the diet. Furthermore, we expect dietary protein content to predict carcass fat content but not carcass protein content, with little difference in carcass protein content across treatments. Thus, the protein content of the diet will predict carcass body composition. Furthermore, we expected carcass fat content to be higher with high lipid intake and low protein intake. For endurance and activity, we predicted that endurance would be greater on high‐protein diets, as protein is important for muscle development while activity would be higher on low‐protein diets to allow protein‐restricted individuals to locate better food sources. Finally, we predicted that testes size would be larger on high‐protein diets.

## 2 METHODS

### 2.1 Husbandry

Experimental individuals were first‐generation offspring of wild‐caught three‐spine sticklebacks. Parents were collected in the spring of 2014 form Inverleith Pond, Edinburgh (55.96N 3.22W). Using standard IVF techniques for this species (Barber & Arnott, 2000), 23 clutches were produced, each with a unique sire and dam. Offspring were fed live *Artemia* until one month of age, after which they were provided live *Artemia* and fry powder (ZM Sytems, ZM‐100 Fry Food: protein 55.0%, oil 13.0% and ash 12.0%) until 3 months of age. From three to four months (the start of dietary manipulations), fish were fed standard‐grade fish pellet (ZM Systems, medium granular: protein 52.0%, oil 12.0% and ash 10.3%) to condition them to surface feeding on fish pellet. At 4 months of age, fish were molecularly sexed from fin clips and weighed. Fish were then randomly assigned to one of five diet treatments, such that an equal number of males and females were assigned to each diet. A total of 150 fish were used, giving 15 fish per sex per diet.

Fish were housed in plastic tanks (30 × 20 × 20 cm), provisioned with an individual air filter and two artificial weeds. Each tank contained three unrelated individuals of the same sex. Individuals were of a different size to enable individual identification of the fish without physically marking them (Lee, Monaghan, & Metcalfe, 2013). Clutches were evenly split between the tanks to control for both tank and family effects. Light and temperature regimes were matched to natural levels in Edinburgh at that time of year.

### 2.2 Diet treatments

Unlike for mice and flies, where most work on macronutrient ratio has been carried out, it has been shown that carbohydrate is not a key macronutrient for predatory fish, with much more importance placed on lipid (Ruohonen et al., 2003). Therefore, we created five diets differing in the ratio of protein:lipid (Table 1). We suggest that in these diets, protein and lipid are not strongly negatively correlated (see Fig. S1), and thus allow us to separate the effect of diet into the independent effects of protein and lipid. To achieve this lack of correlation, we used inert carbohydrate filler, which has been shown to be indigestible in teleosts (Guillaume, 2001; Kim & Kaushik, 1992). Thus, although the diets differ in carbohydrate content (Table 1), this was indigestible to the fish. To test for a correlation between protein and lipid, we use their relative abundance (%) in the raw diet (g). However if you consider the contribution of protein and lipid to usable energy, there is a strong negative correlation (see Table S1). We suggest our approach of considering relative abundance is more appropriate, as we quantify amounts of protein and lipid in body, not energy, and fat will be prioritized as an energy source with protein as a source of structural components, for example, amino acids for growth (see theory of protein sparing: De Silva, Gunasekera, & Shim, 1991; Vergara, Robainà, Izquierdo, & De La Higuera, 1996; Helland & Grisdale‐Helland, 1998 and below). Diets were in pellet form made of different combinations of fish meal and fish oil (Table S2). Diets were manufactured at the Aquaculture and Fish Nutrition Centre (University of Plymouth, Plymouth, U.K.).

**Table 1:** Table of the nutrient content of the five diets used in this experiment. Calories represent the usable energy in the diet, that is, the energy from protein and lipid only, excluding the indigestible carbohydrate. Macronutrient values are percentages of raw materials (g) in the diet (see Table S1 for details of energetic contributions of each nutrient)

Protein (%)	Lipid (%)	Carbohydrate (%)	Ratio P:L	Calories (MJ/kg)
67.5	6.6	15.8	10.2:1	13.8
33.2	3.9	53.1	8.5:1	7.1
59.3	13.0	16.1	4.6:1	14.8
51.6	20.5	17.8	2.5:1	16.3
31.2	19.2	39.7	1.6:1	12.4

In the majority of studies where macronutrients are manipulated, diets are provided ad libitum with food available at all times. However, as food degrades rapidly in water, this feeding regime is not suitable for aquatic organisms. We therefore adapted a previous feeding regime that has been successful in fish (Terzibasi et al., 2009). Here, fish are fed to satiation twice per day, in the morning and in the evening. The amount of food provided for each diet was reassessed monthly, by feeding fish incrementally until satiated. This amount of food was then provided morning and evening for a month until the next reassessment was made. All tanks of the same diet were fed the maximum amount of pellet consumed by any tank on that diet. As a result, the majority of tanks were fed to excess with not all of the food ration being eaten; thus, we cannot quantify how much of the ration was consumed. Therefore, we do not present intake data on an individual or a tank level (e.g., Solon‐Biet et al., 2014). Fish were maintained on diet treatments throughout the course of the experiment (106 days).

### 2.3 Growth and condition

From the start of diet treatments until the end of the study, fish were weighed and length was measured approximately once a month. However, as growth was roughly linear (see Fig. S2), we only analyzed initial weight, to check for any differences between treatments before the start of the experiment, and final weight, to assess differences in growth between diet treatment. Furthermore, a common measure of assessing overall health of a fish is condition index. Here, we calculated condition using residuals from an analysis of the length–weight relationship (see Bentley & Schindler, 2013):

Condition Index=log(Weight)−log(a)−blog(Length)

with the slope (*b*) and intercept (*a*) taken from a model of the log of weight against the log of length for all fish measured in this study (Bentley & Schindler, 2013). A negative value indicates a fish in a poorer than average condition, and a positive value suggests a better than average condition.

### 2.4 Swimming endurance

On one occasion between days 79 and 100, each fish was assessed for their swimming endurance ability. We used the same protocol as described in Alvarez and Metcalfe (2005). Briefly, fish were placed in a swim chamber (length 25 cm, internal diameter 6 cm) submerged in a glass‐sided tank (59 × 29 × 28 cm) filled to a depth of 22 cm with room temperature water. Fish were exposed to two currents, generated within the swim chamber, initially a slow current (4 cm/s) for 5 min, to condition individuals to the swim chamber, after which the speed was increased to 20 cm/s and a timer started. At the first cessation of swimming, fish were prompted to return to swimming by a small tap on the chamber. If this failed to elicit swimming, or at the second refusal to swim, the current and timer were stopped. Where individuals continued to swim, the trial was allowed to run for a maximum of 30 min (5 min acclimatization and 25 min at 20 cm/s). Immediately following the trail, the fish was removed to a recovery tank and a 50% water change performed before another trial was initiated. Temperature was recorded every two hours and then converted into a daily average. Swimming endurance was taken as the time an individual was able to remain swimming while exposed to the high‐speed current, and any fish that swam for the full trial was given a score of 25 min (23 of 118 tested). Swimming endurance tests were performed with the observer blind to dietary treatment.

### 2.5 Activity

To assess the effect of diet on levels of activity, activity trials were conducted between days 79 and 100. Activity trials were carried out in a glass‐sided tank (45 × 25 × 25 cm), containing water to a depth of 8 cm following a similar protocol to Boulton, Grimmer, Rosenthal, Walling, and Wilson (2014). The tank was placed on a light box, surrounded by white walls to prevent disturbance and a video camera mounted above the tank. Each fish was placed in the center of the tank and given a 60‐s acclimatization period, followed by eight‐minute monitoring. Fish activity was tracked using Viewer^3^ tracking software (http://www.biobserve.com/behavioralresearch/products/viewer/). Activity was measured as the total time spent moving during the eight‐minute assessment window. Following the assessment period, the fish was removed and a 100% water change was performed prior to the next trial, thereby ensuring there were no chemical cues remaining in the water which could affect the next trial.

### 2.6 Testes mass

At the end of the experiment (24/02/2015), all males were sacrificed through overdose of tricaine mesylate (MS222) and physical destruction of the brain. They were dried, by blotting with paper towel, and then both testes were removed and transferred to a preweighed Eppendorf. Owing to the delicate nature of the testes, they were not dried prior to weighing. The Eppendorf was then reweighed on a fine balance (±0.001 g), and testes mass was taken as the difference between the two weights (g). Testes measurements were carried out with the observer blind to dietary treatment.

### 2.7 Body composition

On the 25/02/2015, all female fish were also sacrificed through overdose of MS222 and physical destruction of the brain. Carcasses of both sexes were frozen at −20°C until carcass composition analysis was carried out. Wet and dry mass of carcasses were quantified. Soxhlet extraction was used to quantify the fat mass and fat‐free mass (protein mass), and the remaining carcass was then ashed to determine the bone and mineral content of the samples. We therefore quantified body composition as protein content (g), lipid content (g), ash content (g), and the ratio of protein:lipid in the carcass. Analyzing three measures of body composition (ratio of protein:lipid, protein content, and lipid content) allows us to test whether changes in the ratio of macronutrients in the body are driven by variation in protein content, lipid content, or both. Body composition was analyzed blind of the dietary manipulations.

### 2.8 Statistical analysis

All analyses were carried out in R (v3.3.1; R core team, 2016) using the packages *Lme4* (Bates, Mächler, Bolker, & Walker, 2015) and *MCMCglmm* (Hadfield, 2010). Tank and family of origin were included as random effects in all models. The ratio of protein:lipid in the carcass was analyzed via linear mixed effects (LME) models with diet and sex included as categorical fixed effects. Carcass protein, carcass lipid, and carcass ash contents were analyzed via LME models, with diet and sex included as categorical fixed effects and carcass dry weight included as a continuous covariate to account for differences in size. Protein and lipid content of the diets were not strongly negatively correlated (see Fig. S1); therefore, we fitted models to try to separate the effects of dietary protein and lipid. These models included the same fixed and random effects as above, but with dietary protein and lipid included as continuous covariates in place of diet. Testes mass was analyzed via LME with diet as a categorical fixed effect and wet weight included as continuous variable. LME models for wet and dry weight contained diet and sex as categorical fixed effects. To assess the effect of diet on activity, we analyzed total time moving using LME models with diet and sex as factors and wet weight as a covariate. Swimming endurance was analyzed via a Markov chain Monte Carlo generalized linear mixed model (MCMCglmm) using a censored exponential distribution, because this data were exponentially distributed, with a number of fish swimming for the full 25 minutes. To minimize autocorrelation of the model, it was run for 1,300,000 iterations and a burnin of 300,000 with 1,000 samples stored. Diet, sex, wet weight and water temperature were included as fixed effects, and tank was included as a random effect.

## 3 RESULTS

### 3.1 Growth

**Figure 1:**
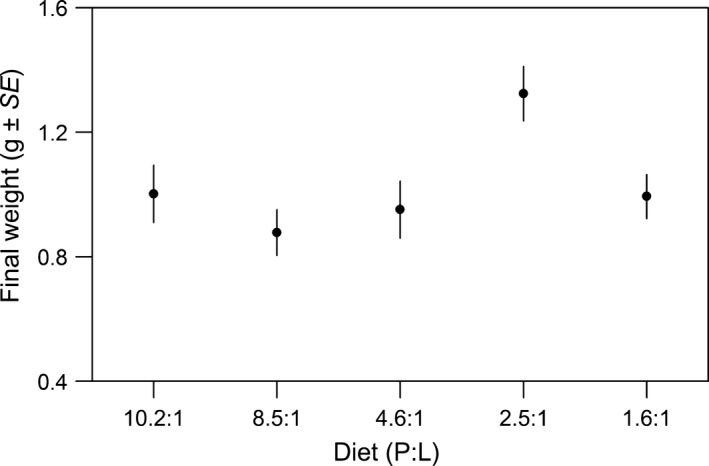
Mean final weight (g ± *SE*) in relation to diet (protein:lipid). There was an effect of diet on final weight (*p* = .001), with individuals on diet 2.5:1 significantly heavier than individuals reared on all other diets (all *p *< .040). There were no differences between the weight of individuals reared on the remaining four diets (all *p *> .6)

There were no significant differences in initial weight or length between the treatments (LME; weight: χ^2^ = 2.11; *p *=* *.716; Fig. S2; length: χ^2^ = 1.33; *p *=* *.857). However, there was a marginally nonsignificant difference between the sexes in initial weight (LME; χ^2^ = 3.38; *p *=* *.066) and a significant effect of sex on initial length (LME; χ^2^ = 4.75; *p *=* *.029), with females being slightly larger than males (mean weight (g) ± *SE*: females 0.43 ± 0.02; males 0.38 ± 0.02; mean length (mm) ± *SE*: females 34.20 ± 0.64; males 32.58 ± 0.58). The marginally nonsignificant difference in initial weight between the sexes disappeared by the final weighing (LME; χ^2^ = 0.98; *p *=* *.323) but remained significant for length at final measuring (LME; χ^2^ = 4.21; *p *=* *.040; mean length (mm) ± *SE*: females 44.60 ± 0.64; males 42.96 ± 0.79). There was a significant effect of diet on final weight (LME; χ^2^ = 18.44; *p *=* *.001; Figure 1) and final length (LME; χ^2^ = 13.43; *p *=* *.009). Post hoc analysis revealed fish on the *2.5:1* diet were significantly heavier than those on all other diets (Table S3), but longer only than fish on the *8.1:1* diet (Table S4, Fig. S3). However, there was no difference in weight or length for all other diet comparisons (Figure 1, post hoc analysis Tables S3 and S4, Fig. S3). Diet also had a significant effect on dry weight (LME; χ^2^ = 28.26; *p *<* *.001), with post hoc analysis again revealing this difference was driven by fish on the *2.5:1* diet being significantly heavier than fish on all other diets (post hoc analysis Table S5). As with wet weight, there was no effect of sex on dry weight of the carcass at the end of the experiment (LME; χ^2^ = 28.26; *p *=* *.197).

As with final weight, there was a significant effect of diet on condition index. However, the pattern of differences between treatments for condition index was not the same as that of weight and length. Fish on the *4.6:1* diet were in significantly poorer body condition than fish on the *8.5:1* and *2.5:1* diets, and a poorer but marginally nonsignificant condition to fish on the *1.6:1* diet (post hoc comparisons Table S6; Figs. S4 and S5). There were no significant differences in condition for all remaining comparisons (Table S6). As with final weight, there was no effect of sex on condition index (*p *=* *.260).

### 3.2 Body composition

**Figure 2:**
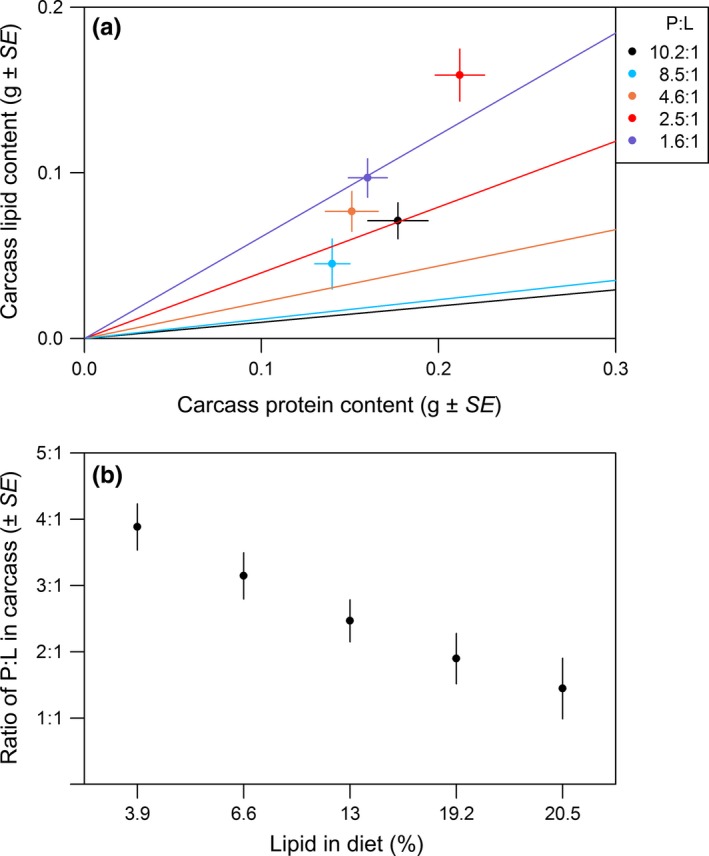
(a) Mean (±*SE*) carcass lipid content (g) against mean (±*SE*) carcass protein content (g). Rails represent the protein:lipid ratios in the five diets. Colors correspond to the five diets (see key). There was a significant effect of diet on the degree of difference between carcass and dietary protein:lipid ratio (*p* < .001). (b) Mean (±*SE*) carcass protein:lipid ratio in relation to dietary lipid (%). Ratio in carcass is carcass protein (g)/carcass lipid (g). Ratio of protein to lipid in the carcass decreased linearly with increasing dietary lipid intake (*p *< .001) but is not significantly affected by protein intake (*p* = .180)

Analysis of the ratio of protein:lipid in the carcass revealed a significant effect of diet (LME; χ^2^ = 38.60; *p *<* *.001; Figure 2; post hoc Table S7). Interestingly, the protein:lipid ratio in the carcass did not match that of the diet, nor show the same rank order. The ratio of protein:lipid was lower in the fish than in the diet that they had consumed, with the biggest difference in fish from the highest protein:lipid diet (Figure 2a). To test this, we analyzed the difference between the protein:lipid ratio of the diet and that of the carcass of fish fed on that diet. There was indeed a significant effect of diet. Fish fed on high protein:lipid ratio diets had more of a difference between their body composition and the composition of the diet than fish fed on lower protein:lipid ratio diets (LME; χ^2^ = 118.59; *p *<* *.001; post hoc analysis Table S8; Fig. S6).

Investigating the effect of the protein and lipid content of the diet separately revealed that the carcass protein:lipid ratio was significantly linearly influenced by the percentage of lipid in the diet (LME; χ^2^ = 37.16; *p *<* *.001), but not the percentage of protein (LME; χ^2^ = 1.79; *p *=* *.180; Fig. S7), with the protein:lipid ratio of the carcass decreasing with increasing lipid content of the diet (Figure 2b). Carcass protein:lipid ratio also differed between the sexes (LME; χ^2^ = 4.54; *p *=* *.033), with males having a lower ratio than females (mean ratio of protein:lipid ± *SE*: males 2.3:1 ± 0.1, females 2.9:1 ± 0.2).

Similar patterns were observed when independently analyzing the protein and lipid content of the carcass rather than their ratio. Diet had a significant effect on both protein (LME; χ^2^ = 53.06; *p *<* *.001; post hoc analysis Table S9) and lipid content (LME; χ^2^ = 42.59; *p *<* *.001; post hoc analysis Table S10) of the carcass when controlling for variation in dry weight (LME: Protein: χ^2^ = 381.52; *p *<* *.001. Lipid: χ^2^ = 261.91; *p *<* *.001), with protein content of the carcass increasing and lipid content decreasing as the dietary ratio of protein:lipid increased (Fig. S8). However, as with carcass protein:lipid ratio, this was driven by a linear effect of dietary lipid content, rather than an effect of dietary protein content: There was a negative linear effect of dietary lipid on carcass protein and a positive effect on carcass lipid (LME; Carcass protein χ^2^ = 38.23; *p *<* *.001; Carcass lipid χ^2^ = 37.50; *p *<* *.001; respectively; Fig. S8), but no effect of dietary protein (LME: Carcass protein χ^2^ = 0.28; *p *=* *.600; Carcass lipid χ^2^ = 0.17; *p *=* *.677; Fig. S8). Finally, there was a significant effect of sex on carcass lipid content (LME; χ^2^ = 7.76; *p *=* *.005), with males having greater lipid content of the carcass (mean lipid content (%) ± *SE*: males 28.09 ± 1.10, females 24.72 ± 1.20). However, the effect of sex was marginally nonsignificant for protein content (LME; χ^2^ = 3.68; *p *=* *.055), suggesting that ash content must differ. We therefore analyzed ash content, which is a measure of carcass bone and mineral content. There was a significant effect of sex on ash content (LME; χ^2^ = 5.00; *p *=* *.025), with females having greater ash than males (mean ash content (%) ± *SE*: males 15.09 ± 0.63, females 16.91 ± 0.63).

### 3.3 Testes mass

There was a positive linear effect of final weight on testes mass (LME; χ^2^ = 13.17; *p *<* *.001; estimate ± *SE* (g): 0.00401 ± 0.00111). Accounting for final weight, there was no effect of diet on testes mass (LME; χ^2^ = 3.96; *p *=* *.412). However, despite the effect of diet on final weight, there was no evidence of an indirect effect of diet on testes mass, as diet was still nonsignificant when final weight was excluded from the model (LME; diet: χ^2^ = 0.864; *p *=* *.930).

### 3.4 Swimming endurance and activity

The censored exponential model revealed no significant effect of diet, sex, weight, or water temperature on swimming endurance (MCMCglmm; all *p *>* *.08; Table S11). To assess activity, we analyzed total time spent moving during the eight‐minute assessment window. This revealed no significant effect of diet, sex, or weight on activity level (LME; Diet: χ^2^ = 3.07; *p *=* *.547; Sex: χ^2^ = 0.691; *p *=* *.406; Weight: χ^2^ = 0.844; *p *=* *.358; Table S12).

## 4 DISCUSSION

Diet is known to be an important determinant of key fitness traits (Fontana & Partridge, 2015; Partridge et al., 2005). However, what mediates this effect is much less well understood. Our study explores the relationship between dietary macronutrient ratio and the macronutrient composition of the body, a key determinant of fitness traits such as health and lifespan. In particular, we explore the direct effect of dietary protein and lipid intake on protein and lipid content in the body. Interestingly, our findings suggest that individuals are able to alter their utilization or uptake of ingested macronutrients, with the ratio of protein:lipid in the carcass being vastly different from that of the diet. Furthermore, we found no effect of dietary protein intake on body composition, rather carcass protein and lipid content was predicted only by dietary lipid intake. Although the protein leverage hypothesis focuses on protein intake, these results conflict with our predicted outcomes of this for body composition (Simpson & Raubenheimer, 2005). Under the protein leverage hypothesis, we expected the rank order of diet protein:lipid ratios to be maintained in the ratio of protein:lipid in the carcass. Furthermore, we expected that the protein content of the diet would predict carcass body composition and the relative protein content of the body would be relatively stable. However here, there was no effect of protein intake on body composition, the rank order of protein:lipid ratios was not maintained from the diet to the carcass, and the protein content of the body varied across diets.

These findings have striking implications for studies exploring the relationship between diet and health or organismal fitness. It has been suggested that being consigned to a specific diet, but fed ad lib, allows individuals to increase or decrease their intake of that diet, but prevents them from altering the ratio of macronutrients they ingest (Simpson & Raubenheimer, 1995, 2007; Simpson, Sibly, Lee, Behmer, & Raubenheimer, 2004). However, our results show individuals clearly alter their utilization or uptake of the ingested macronutrients, resulting in vastly different macronutrient ratios in the carcass compared to the body. Furthermore, the range of protein:lipid ratios was 1.4:1 to 3.9:1 in the carcasses, but was 1.6:1 to 10.2:1 in the diets. This suggests a pattern of modification toward a lower and less variable carcass protein:lipid ratio. Previous work has suggested that lifespan is maximized on low protein:nonprotein intakes, with high‐protein diets negatively affecting lifespan (Carey et al., 2008; Fanson et al., 2009; Jensen et al., 2015; Lee et al., 2008; Maklakov et al., 2008), which could imply that individuals are targeting lower protein:nonprotein ratios in an attempt to increase fitness.

Previous research suggests a survival cost to being maintained on an imbalanced diet. Two obvious alternative explanations for this are the cost of storage of excess nutrients or the cost of their selective absorption or excretion. Our results provide some support for the latter. Individuals fed diets of vastly different macronutrient ratios appeared to converge on more similar body compositions. This suggests that nutrients are not simply stored in proportion to their availability in the diet and thus that survival costs of imbalanced diets are likely associated with selective absorption or excretion of particular nutrients. Given that here, dietary lipid content, not protein, is driving body composition and the positive association between dietary lipid intake and adiposity (Hariri & Thibault, 2010), we suggest that this modification is achieved via metabolism of excess protein. The body has a limited capacity for storing excess protein, which must be converted into urea and excreted (Delimaris, 2013; Heaney, 1998; Tarnopolsky et al., 1992) which may represent one potential cost of a high‐protein diet (Fanson et al., 2012).

Our results also provide mixed support for the well‐known theory of protein sparing in fish, where individuals prioritize lipid use for energy expenditure and use protein for growth and muscle development (De Silva et al., 1991; Helland & Grisdale‐Helland, 1998; Vergara et al., 1996). The lack of an effect of protein content of the diet on protein content of the carcass suggests individual fish were able to maintain the protein content of their carcass on protein intakes as low as 31.2% and conforms to the theory of protein sparing. However, the negative linear effect of lipid intake on carcass protein content is counter to predictions from protein sparing.

There was little effect of diet on growth, despite diets of differing energy levels being well known to affect size (e.g., Colman et al., 1998; McCay et al., 1935). However, in our study, food was provided ad libitum, meaning that despite the diets differing in energy content (Table 1), fish on lower energy diets could increase their intake and avoid caloric restriction. Only fish on the *2.5:1* diet were different in size, being significantly larger than all other fish in all other diets. Interestingly, the protein:lipid ratio in this diet is closest to the ratio that maximizes growth in European Whitefish, *Coregonus lavaretus* (Ruohonen et al., 2003). Ruohonen et al. (2003) suggested that growth was maximized on a 2.25:1 protein:lipid ratio as this feed had a high energy value. However, this explanation is unlikely here, as food was provided ad lib (see above), and there were no differences in growth between other diets differing greatly in energy content (e.g., 7.1 MJ/kg to 14.8 MJ/kg). We suggest that the *2.5:1* diet resulted in the greatest growth because it had the highest energy content in combination with a balance of protein and lipid and that high levels of no single dietary component can generate high levels of growth.

Our results also provide evidence of sexual dimorphism in body composition, with males being significantly shorter and having greater fat deposits, and females being longer and having higher bone and mineral deposits (indicated by the higher ash content). These findings fit with a previous study (Kitano, Mori, & Peichel, 2007), where female *G. aculeatus* were also found to be longer than males. We suggest that this is likely a result of the different reproductive behaviors exhibited by the sexes. When reproducing, male three‐spine sticklebacks defend territories, construct nests, court females, and fan eggs, which likely impacts on their ability to forage (Wootton, 1984). Therefore, males potentially invest in fat deposition, rather than growth in length, to provide greater energy reserves prior to the breeding season. This would explain the higher fat content of males here, as our fish were culled immediately prior to the breeding season.

We found no effect of diet on swimming endurance or activity, despite calorie restriction being known to affect activity and endurance (reviewed Speakman & Mitchell, 2011). However, individuals in the current study were fed ad libitum and could therefore obtain sufficient energy to maintain activity levels. Additionally, as discussed above, fish appeared able to selectively utilize their ingested macronutrients and therefore may not have been under major macronutrient imbalance; thus, there was no stimulation to increase activity levels. Alternatively, these findings could suggest that the effects of calorie restriction on performance are not reproducible through macronutrient manipulations. It is also possible that any differences in activity and endurance were too subtle to be detected in the current study.

Finally, we found no direct or indirect effect of diet on testes mass. This could reinforce the suggestion that the testes are protected from the effect of diet (Mitchell et al., 2015). Alternatively, it could suggest that testes size in the three‐spine stickleback is a low‐cost reproductive trait, and thus that the effect of diet is correspondingly small and therefore difficult to detect (Moatt et al., 2016).

In conclusion, we show that body macronutrient composition differs from that of the diet and that this pattern of variation suggests individuals are attempting to achieve a particular protein:lipid ratio in the body rather than prioritising a single macronutrient. We suggest individuals are achieving a balance of protein and lipid in the body by excreting excess protein. In contrast to a number of recent studies and the protein leverage hypothesis (Huang et al., 2013; Lee, 2015; Ponton et al., 2015; Skorupa et al., 2008; Solon‐Biet et al., 2015; Sørensen et al., 2008), our results suggest lipid intake is the key determinant of body composition, rather than protein. Together, these data suggest that the key macronutrient for determining body composition may differ between species, which, if this extends to lifespan, has striking implications for studies of DR, where effects have been suggested to be evolutionarily conserved (e.g., see Moatt et al., 2016; Nakagawa et al., 2012). The results presented here seem to conflict with predicted outcomes of the protein leverage hypothesis, but we do not directly quantify intakes of either protein or lipid. Given that the protein leverage hypothesis directly relates to intake, it would be interesting to examine the intake of the diets used here and see if they match the patterns observed for body composition. Future studies should also look to test whether a particular body composition is achieved via protein excretion and whether the costs of excreting protein could be one explanation for the emerging survival cost of being maintained on a high‐protein diet (Fanson et al., 2012).

## DATA ACCESSIBILITY

The datasets and materials analysed during the current study are available in the Dryad Repository, doi: 10.5061/dryad.k3rv4.

## CONFLICT OF INTEREST

None declared.

## AUTHOR CONTRIBUTIONS

JPM and CAW conceived and designed the study. Data collection was carried out by JPM, EH, FM, and AK. Body composition analysis was carried out by CH and JRS at the University of Aberdeen. Statistical analysis was carried out by JPM and CAW. JPM wrote the initial draft of the manuscript, and CAW and JPM performed revisions. All authors approved the final version of the manuscript.
