# Supplementary material for: Body macronutrient composition is predicted by lipid and not protein content of the diet
Source: Ecol Evol. 2017 Oct 22;7(23):10056–65. doi: 10.1002/ece3.3529 (PMC5723615; doi:10.1002/ece3.3529)
Supplement: Supplementary file 1 [file ECE3-7-10056-s001.docx]

Appendix 1: supplementary figures and tables for the article:

Body macronutrient composition is predicted by lipid and not protein content of the diet.

Joshua P. Moatt^*a^, Catherine Hambly^b^, Elizabeth Heap^c^, Anna Kramer^a^, Fiona Moon^a^, John R. Speakman^b,d^ & Craig A. Walling^a^

^a^ Institute of Evolutionary Biology, School of Biological Sciences, University of Edinburgh, Edinburgh, UK.

^b^ Institute of Biological and Environmental Sciences, University of Aberdeen, Aberdeen, UK.

^c^ Edinburgh Genomics, Roslin Institute, University of Edinburgh, Edinburgh, UK.

^d^ State Key Laboratory of Molecular Developmental Biology, Institute of Genetics and Developmental Biology, Chinese Academy of Sciences, China.

* Corresponding Author: josh.moatt@ed.ac.uk / joshmoatt@gmail.com

Supplementary materials.

**Supplementary Tables**

**Table S1.** Energy contribution from protein and lipid in each of the 5 diets used in this study. Useable calories represents the energy available from digestible components of the diet (i.e. protein and lipid only). When considering the raw amounts of protein and lipid in the diets, protein and lipid do not negatively correlate (see supplementary Fig. S1). However, when considering them in terms of their contribution to usable energy (%) there is a perfect negative correlation.

| Ratio P:L | Protein (%) | Protein Energy (MJ/kg) | Lipid (%) | Lipid Energy (MJ/kg) | Useable Calories (MJ/kg) | Useable Energy (%) from Protein | Useable Energy (%) from Lipid |
| --- | --- | --- | --- | --- | --- | --- | --- |
| 10.2 : 1 | 67.5 | 11.3 | 6.6 | 2.5 | 13.8 | 81.9 | 18.1 |
| 8.5 : 1 | 33.2 | 5.6 | 3.9 | 1.5 | 7.1 | 78.9 | 21.1 |
| 4.6 : 1 | 59.3 | 9.9 | 13.0 | 4.9 | 14.8 | 66.9 | 33.1 |
| 2.5 : 1 | 51.6 | 8.6 | 20.5 | 7.7 | 16.3 | 52.8 | 47.2 |
| 1.6 : 1 | 31.2 | 5.2 | 19.2 | 7.2 | 12.4 | 41.9 | 58.1 |

**Table S2.** Recipe for the five diets used in this experiment. Herring meal is both a source of protein and lipid, therefore fish oil was only required in diets with high lipid contents.

| Ingredient (%) | 10.2 : 1 | 8.5 : 1 | 4.6 : 1 | 2.5 : 1 | 1.6 : 1 |
| --- | --- | --- | --- | --- | --- |
| Herring Meal | 90.24 | 41.65 | 79.83 | 69.42 | 41.65 |
| Corn Starch (Filler) | 6.26 | 54.85 | 11.17 | 13.61 | 38.77 |
| Lecithin | 1.00 | 1.00 | 1.00 | 1.00 | 1.00 |
| Vitamin /mineral premix | 1.00 | 1.00 | 1.00 | 1.00 | 1.00 |
| ASTX (10% carophyll pink) | 1.00 | 1.00 | 1.00 | 1.00 | 1.00 |
| CMC binder | 0.50 | 0.50 | 0.50 | 0.50 | 0.50 |
| Fish Oil | 0.00 | 0.00 | 5.50 | 13.47 | 16.08 |

**Table S3.** Output from post hoc Tukey analysis of model exploring the effect of diet on the final weight of fish.

| Comparison | Estimate. (s.e.) | z | *p* |
| --- | --- | --- | --- |
| *4.6 : 1* – *10.2 : 1* | -0.061 (0.111) | -0.543 | 0.983 |
| *2.5 : 1* – *10.2 : 1* | 0.317 (0.113) | 2.802 | 0.040 |
| *8.5 : 1* – *10.2 : 1* | -0.140 (0.104) | -1.353 | 0.657 |
| *1.6 : 1* – *10.2 : 1* | -0.005 (0.106) | -0.049 | > 0.999 |
| *2.5 : 1* – *4.6 : 1* | 0.377 (0.115) | 3.273 | 0.010 |
| *8.5 : 1* – *4.6 : 1* | -0.080 (0.106) | -0.751 | 0.944 |
| *1.6 : 1* – *4.6 : 1* | 0.055 (0.109) | 0.508 | 0.987 |
| *8.5 : 1* – *2.5 : 1* | -0.457 (0.108) | -4.233 | < 0.001 |
| *1.6 : 1* – *2.5 : 1* | -0.322 (0.111) | -2.907 | 0.030 |
| *1.6 : 1* – *8.5 : 1* | 0.135 (0.101) | 1.331 | 0.671 |

**Table S4.** Output from post hoc Tukey analysis of model exploring the effect of diet on the final length of fish.

| Comparison | Estimate. (s.e.) | z | *p* |
| --- | --- | --- | --- |
| *4.6 : 1* – *10.2 : 1* | 0.225 (1.594) | 0.141 | > 0.999 |
| *2.5 : 1* – *10.2 : 1* | 3.292 (1.615) | 2.038 | 0.247 |
| *8.5 : 1* – *10.2 : 1* | -2.399 (1.484) | -1.617 | 0.486 |
| *1.6 : 1* – *10.2 : 1* | -0.120 (1.523) | -0.079 | > 0.999 |
| *2.5 : 1* – *4.6 : 1* | 3.067 (1.649) | 1.860 | 0.338 |
| *8.5 : 1* – *4.6 : 1* | -2.625 (1.519) | -1.727 | 0.416 |
| *1.6 : 1* – *4.6 : 1* | -0.347 (1.558) | -0.222 | 0.999 |
| *8.5 : 1* – *2.5 : 1* | -5.692 (1.545) | -3.684 | 0.002 |
| *1.6 : 1* – *2.5 : 1* | -3.413 (1.583) | -2.155 | 0.196 |
| *1.6 : 1* – *8.5 : 1* | 2.279 (1.452) | 1.570 | 0.516 |

**Table S5.** Output from post hoc Tukey analysis of model exploring the effect of diet on carcass dry weight.

| Comparison | Estimate. (s.e.) | z | *p* |
| --- | --- | --- | --- |
| *4.6 : 1* – *10.2 : 1* | -0.022 (0.037) | -0.585 | 0.977 |
| *2.5 : 1* – *10.2 : 1* | 0.133 (0.038) | 3.515 | 0.004 |
| *8.5 : 1* – *10.2 : 1* | -0.066 (0.035) | -1.886 | 0.324 |
| *1.6 : 1* – *10.2 : 1* | 0.006 (0.036) | 0.153 | > 0.999 |
| *2.5 : 1* – *4.6 : 1* | 0.155 (0.038) | 4.053 | < 0.001 |
| *8.5 : 1* – *4.6 : 1* | -0.045 (0.036) | -1.251 | 0.721 |
| *1.6 : 1* – *4.6 : 1* | 0.027 (0.037) | 0.752 | 0.944 |
| *8.5 : 1* – *2.5 : 1* | -0.120 (0.036) | -5.519 | < 0.001 |
| *1.6 : 1* – *2.5 : 1* | -0.128 (0.037) | -3.442 | 0.005 |
| *1.6 : 1* – *8.5 : 1* | 0.072 (0.034) | -2.091 | 0.224 |

**Table S6.** Output from post hoc Tukey analysis of model exploring the effect of diet on condition index.

| Comparison | Estimate. (s.e.) | z | *p* |
| --- | --- | --- | --- |
| *4.6 : 1* – *10.2 : 1* | -0.096 (0.045) | -1.779 | 0.207 |
| *2.5 : 1* – *10.2 : 1* | 0.075 (0.046) | 1.640 | 0.482 |
| *8.5 : 1* – *10.2 : 1* | 0.040 (0.042) | 0.938 | 0.881 |
| *1.6 : 1* – *10.2 : 1* | 0.024 (0.043) | 0.469 | 0.981 |
| *2.5 : 1* – *4.6 : 1* | 0.171 (0.047) | 3.303 | 0.003 |
| *8.5 : 1* – *4.6 : 1* | 0.136 (0.043) | 2.765 | 0.015 |
| *1.6 : 1* – *4.6 : 1* | 0.120 (0.044) | 2.274 | 0.051 |
| *8.5 : 1* – *2.5 : 1* | -0.035 (0.044) | -0.808 | 0.934 |
| *1.6 : 1* – *2.5 : 1* | -0.050 (0.045) | -1.225 | 0.796 |
| *1.6 : 1* – *8.5 : 1* | -0.016 (0.041) | -0.472 | 0.996 |

**Table S7.** Output from post hoc Tukey analysis of model exploring the effect of diet on the ratio of Protein : Lipid in the carcass.

| Comparison | Estimate. (s.e.) | z | *p* |
| --- | --- | --- | --- |
| *4.6 : 1* – *10.2 : 1* | -0.692 (0.377) | -1.835 | 0.353 |
| *2.5 : 1* – *10.2 : 1* | -1.653 (0.382) | -4.320 | < 0.001 |
| *8.5 : 1* – *10.2 : 1* | 0.697 (0.358) | 1.946 | 0.292 |
| *1.6 : 1* – *10.2 : 1* | -1.251 (0.365) | -3.432 | 0.005 |
| *2.5 : 1* – *4.6 : 1* | -0.961 (0.387) | -2.481 | 0.095 |
| *8.5 : 1* – *4.6 : 1* | 1.389 (0.363) | 3.830 | 0.001 |
| *1.6 : 1* – *4.6 : 1* | -0.560 (0.369) | -1.515 | 0.552 |
| *8.5 : 1* – *2.5 : 1* | 2.350 (0.370) | 6.354 | < 0.001 |
| *1.6 : 1* – *2.5 : 1* | 0.402 (0.375) | 1.071 | 0.821 |
| *1.6 : 1* – *8.5 : 1* | -1.949 (0.350) | -5.567 | < 0.001 |

**Table S8.** Output from post hoc Tukey analysis of model exploring the effect of diet on the difference in Protein : Lipid content between diet and carcass, i.e. degree of change in Protein : Lipid.

| Comparison | Estimate. (s.e.) | z | *p* |
| --- | --- | --- | --- |
| *4.6 : 1* – *10.2 : 1* | 4.978 (0.377) | 13.190 | < 0.001 |
| *2.5 : 1* – *10.2 : 1* | 6.061 (0.383) | 15.825 | < 0.001 |
| *8.5 : 1* – *10.2 : 1* | 2.412 (0.359) | 6.735 | < 0.001 |
| *1.6 : 1* – *10.2 : 1* | 7.355 (0.365) | 20.154 | < 0.001 |
| *2.5 : 1* – *4.6 : 1* | 1.083 (0.388) | 2.792 | 0.041 |
| *8.5 : 1* – *4.6 : 1* | -2.563 (0.363) | -7.058 | < 0.001 |
| *1.6 : 1* – *4.6 : 1* | 2.377 (0.370) | 6.430 | < 0.001 |
| *8.5 : 1* – *2.5 : 1* | -3.645 (0.370) | -9.845 | < 0.001 |
| *1.6 : 1* – *2.5 : 1* | 1.294 (0.375) | 3.448 | 0.005 |
| *1.6 : 1* – *8.5 : 1* | 4.939 (0.350) | 14.097 | < 0.001 |

**Table S9.** Output from post hoc Tukey analysis of model exploring the effect of diet on protein content of carcass, with dry weight included in the model.

| Comparison | Estimate. (s.e.) | z | *p* |
| --- | --- | --- | --- |
| *4.6 : 1* – *10.2 : 1* | -0.017 (0.003) | -4.394 | < 0.001 |
| *2.5 : 1* – *10.2 : 1* | -0.033 (0.004) | -8.066 | < 0.001 |
| *8.5 : 1* – *10.2 : 1* | -0.004 (0.004) | -1.207 | 0.747 |
| *1.6 : 1* – *10.2 : 1* | -0.020 (0.004) | -5.502 | < 0.001 |
| *2.5 : 1* – *4.6 : 1* | -0.016 (0.004) | -3.851 | 0.001 |
| *8.5 : 1* – *4.6 : 1* | 0.012 (0.004) | 3.344 | 0.007 |
| *1.6 : 1* – *4.6 : 1* | -0.003 (0.004) | -0.930 | 0.885 |
| *8.5 : 1* – *2.5 : 1* | 0.028 (0.004) | 6.822 | < 0.001 |
| *1.6 : 1* – *2.5 : 1* | 0.013 (0.004) | 3.167 | 0.013 |
| *1.6 : 1* – *8.5 : 1* | -0.016 (0.004) | -4.408 | < 0.001 |

**Table S10.** Output from post hoc Tukey analysis of model exploring the effect of diet on lipid content of carcass, with dry weight included in the model.

| Comparison | Estimate. (s.e.) | z | *p* |
| --- | --- | --- | --- |
| *4.6 : 1* – *10.2 : 1* | 0.013 (0.005) | 2.396 | 0.117 |
| *2.5 : 1* – *10.2 : 1* | 0.034 (0.006) | 6.057 | < 0.001 |
| *8.5 : 1* – *10.2 : 1* | -0.000 (0.005) | -0.033 | 1.000 |
| *1.6 : 1* – *10.2 : 1* | 0.023 (0.005) | 4.588 | < 0.001 |
| *2.5 : 1* – *4.6 : 1* | 0.021 (0.006) | 3.719 | 0.002 |
| *8.5 : 1* – *4.6 : 1* | -0.013 (0.005) | -2.530 | 0.084 |
| *1.6 : 1* – *4.6 : 1* | 0.011 (0.005) | 2.070 | 0.232 |
| *8.5 : 1* – *2.5 : 1* | -0.034 (0.006) | -6.017 | < 0.001 |
| *1.6 : 1* – *2.5 : 1* | -0.011 (0.005) | -1.952 | 0.289 |
| *1.6 : 1* – *8.5 : 1* | 0.023 (0.005) | 4.783 | < 0.001 |

**Table S11** Output from censored exponential MCMCglmm model. All results are non-significant.

|  | Posterior mean | | Lower 95% CI | Upper 95% CI | Effective sample size | pMCMC |
| --- | --- | --- | --- | --- | --- | --- |
| Intercept | | 225.540 | -193.348 | 663.043 | 1000 | 0.342 |
| *8.5:1* | | 65.665 | -45.209 | 207.681 | 1000 | 0.314 |
| *4.6:1* | | 41.635 | -82.533 | 180.794 | 1000 | 0.542 |
| *2.5:1* | | 51.654 | -66.268 | 173.139 | 1000 | 0.386 |
| *1.6:1* | | 113.842 | -10.893 | 229.301 | 1000 | 0.080 |
| Weight | | -1.771 | -9.194 | 6.871 | 1000 | 0.654 |
| Sex (male) | | -18.969 | -97.846 | 50.116 | 1000 | 0.628 |
| Temperature | | -24.466 | -69.437 | 19.413 | 1000 | 0.300 |

nitt = 1,300,000; thin = 1,000; burnin = 300,000

**Table S12.** Estimate (s.e.) from analysis of activity (total time active).

|  | Estimate (s.e.) |
| --- | --- |
| Intercept | 17.203 (3.687) |
| *8.5 : 1* | 1.337 (2.671) |
| *4.6 : 1* | 0.191 (2.532) |
| *2.5 : 1* | 0.037 (2.560) |
| *1.6 : 1* | 3.696 (2.577) |
| Sex (male) | -1.387 (1.746) |
| Weight | -3.166 (3.559) |

Supplementary Figures


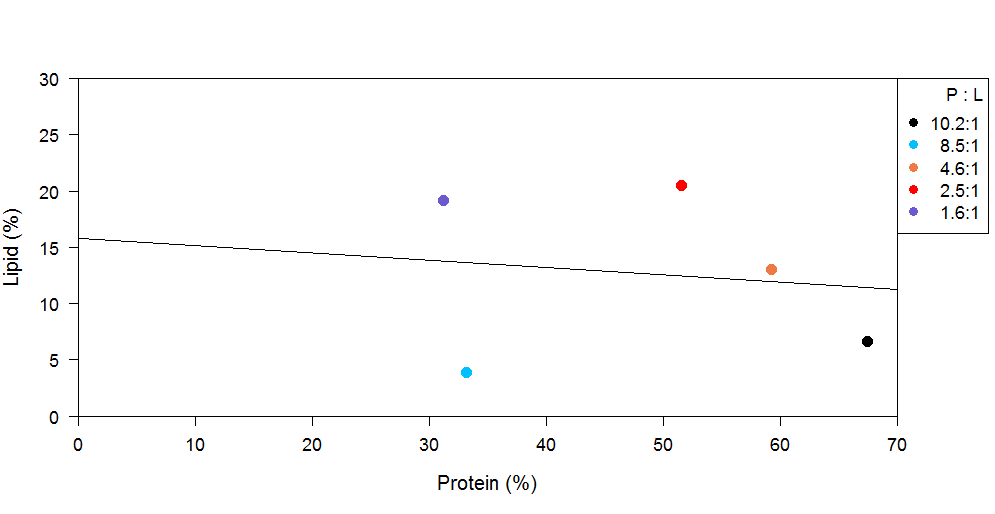


**Figure S1.** The relationship between the lipid (%) and protein (%) contents of the five diets. Colours indicate the diet (see key). The black line represents the regression line from a linear model of lipid content against protein content (slope = -0.0649±0.264). Pearson’s correlation analysis shows protein and lipid are not strongly negatively correlated in the diets (Pearson’s correlation = -0.141 (95% confidence interval = -0.910 to 0.847), t_3_ = -0.246, p = 0.822).


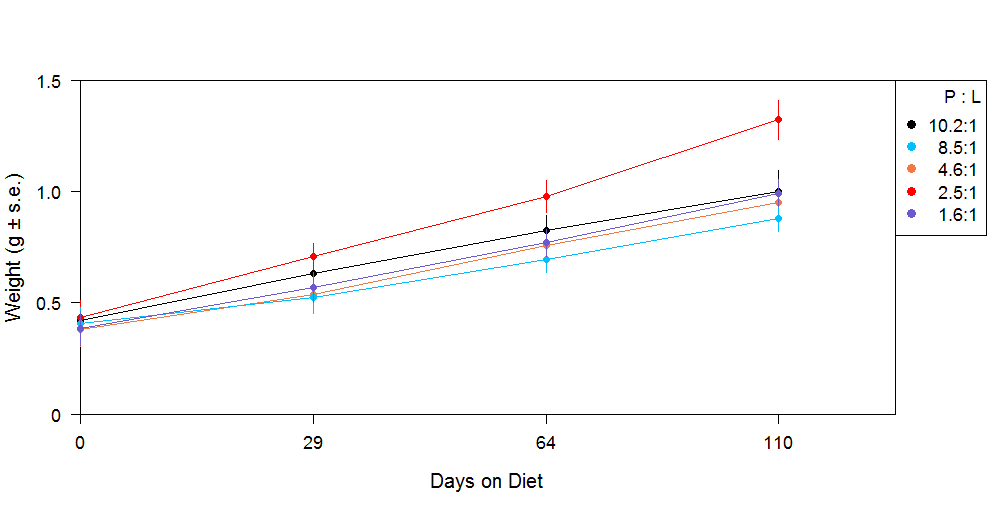


**Figure S2.** Mean weight (± s.e.) in relation to the number of days on the diet treatments. Colours indicate diets (see key). There was no difference between diet treatments initially (p = 0.716). However there was a significant effect of diet on final weight (*p* = 0.001), where *2.5:1* diet is significantly different from all other diets (all *p* < 0.040).


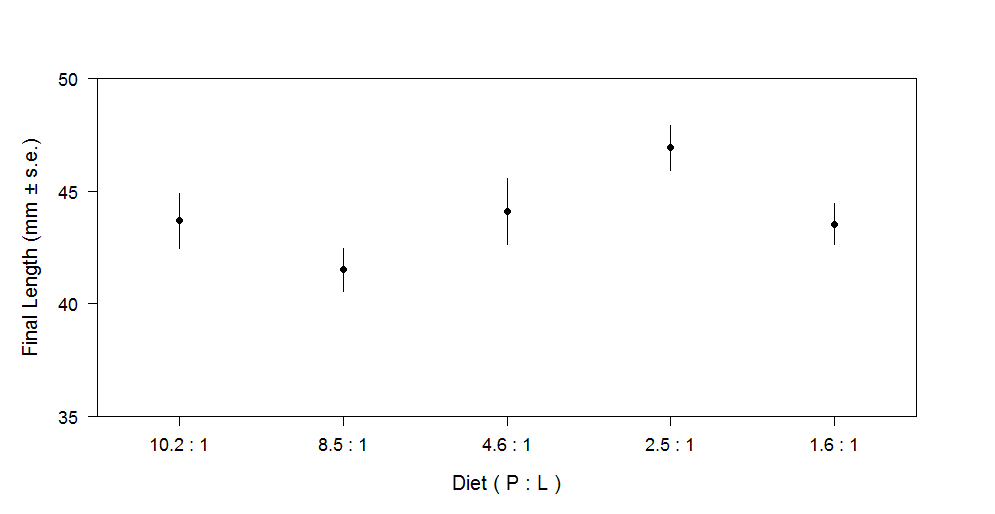


**Figure S3.** Final length of fish (mm ± s.e.) in relation to diet (protein : lipid). There was a significant effect of diet (*p* = 0.009). However, the only significant difference was between the 8*.5 : 1* and 2.5 *: 1* diets (*p* = 0.002). All other comparisons were non-significant (*p* > 0.1).


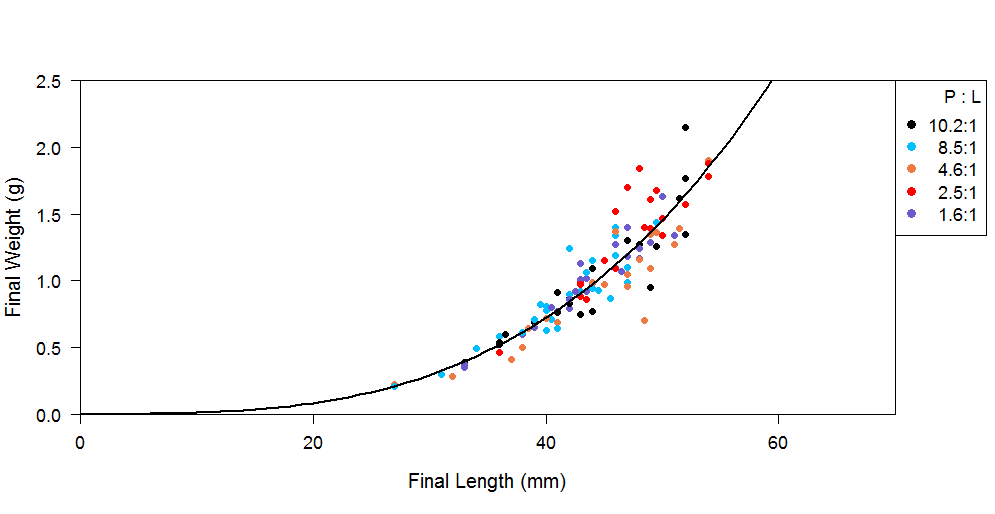


**Figure S4.** Final length of fish against final weight of fish. You can see the expected non-linear relationship between weight and length. Colours correspond to the five diets (see key). The black line represents the predicted weight for fish, calculated as above. Points above this line have a positive condition index, points below have a negative condition index.


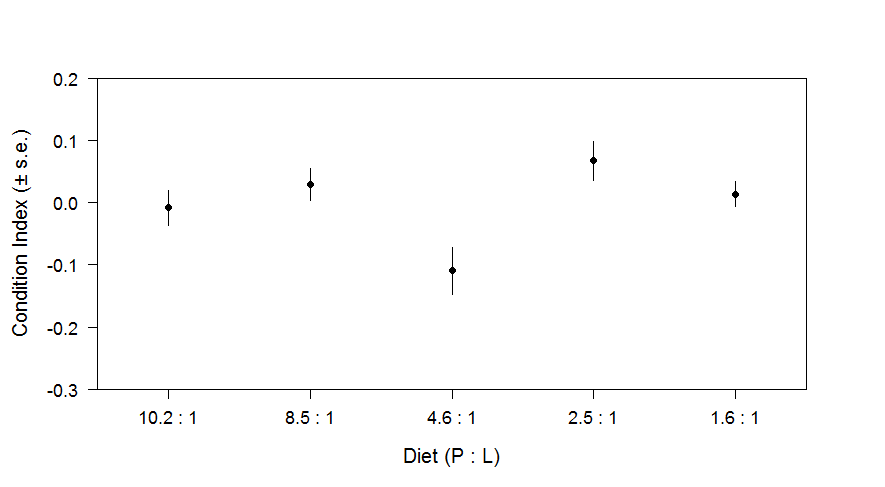


**Figure S5.** Mean condition index of fish (± s.e.) in relation to diet (protein : lipid). A positive condition index indicates a better than average condition, a negative value suggests poorer than average condition. There was a significant effect of diet (*p* =0.014), which was driven by fish on the *4.6 : 1* diet having a lower condition index. The difference was significant when compared to the *2.5 : 1* (*p =* 0.009) and *8.5 : 1* (*p* = 0.045) diets and a marginally non-significant difference when compared to the *1.6*:*1* diet (*p* = 0.051). All other comparisons were non-significant (all *p* > 0.2). There was also no effect of sex (*p* = 0.260).


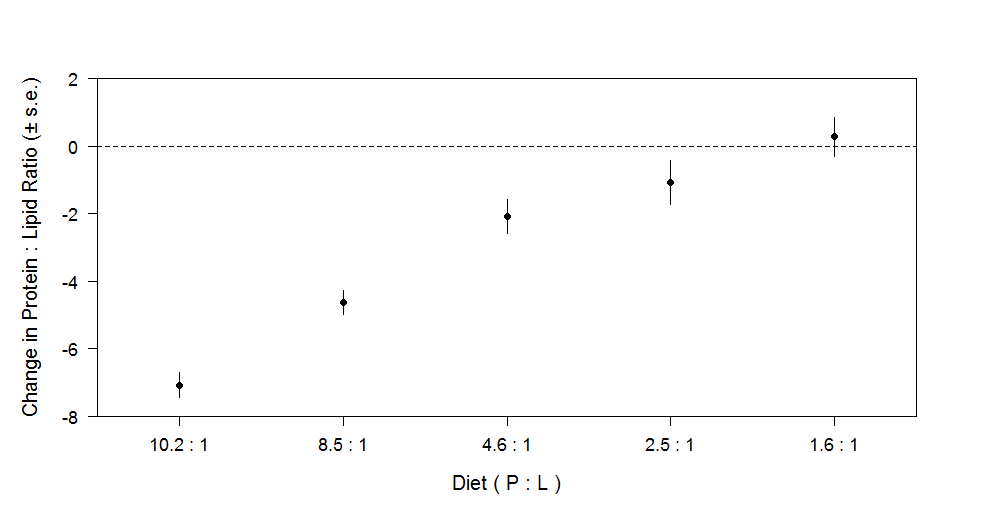


**Figure S6.** Mean difference between the dietary protein : lipid ratio and carcass protein : lipid ratio (± s.e.). The dashed line represents zero, or no difference in the protein : lipid ratio of the diet compared to that of the carcass. The change in protein : lipid was significantly different between all the diets (all *p* < 0.041).


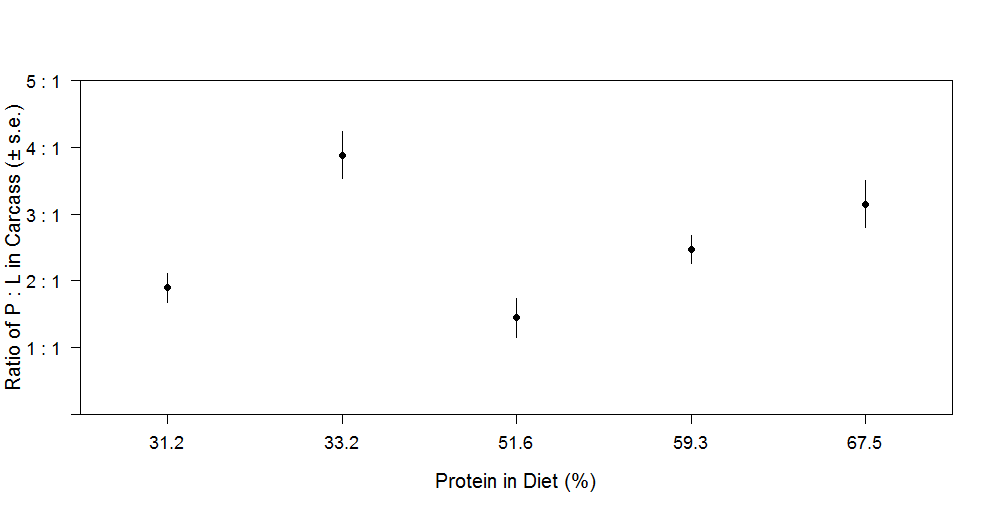


**Figure S7.** Mean (± s.e.) carcass protein : lipid ratio in relation to dietary protein (%).Ratio in carcass is carcass protein (g) / carcass lipid (g). Ratio of protein to lipid in the carcass decreased linearly with increasing dietary lipid intake (*p* < 0.001), but is not significantly affected by protein intake (p = 0.180).

**
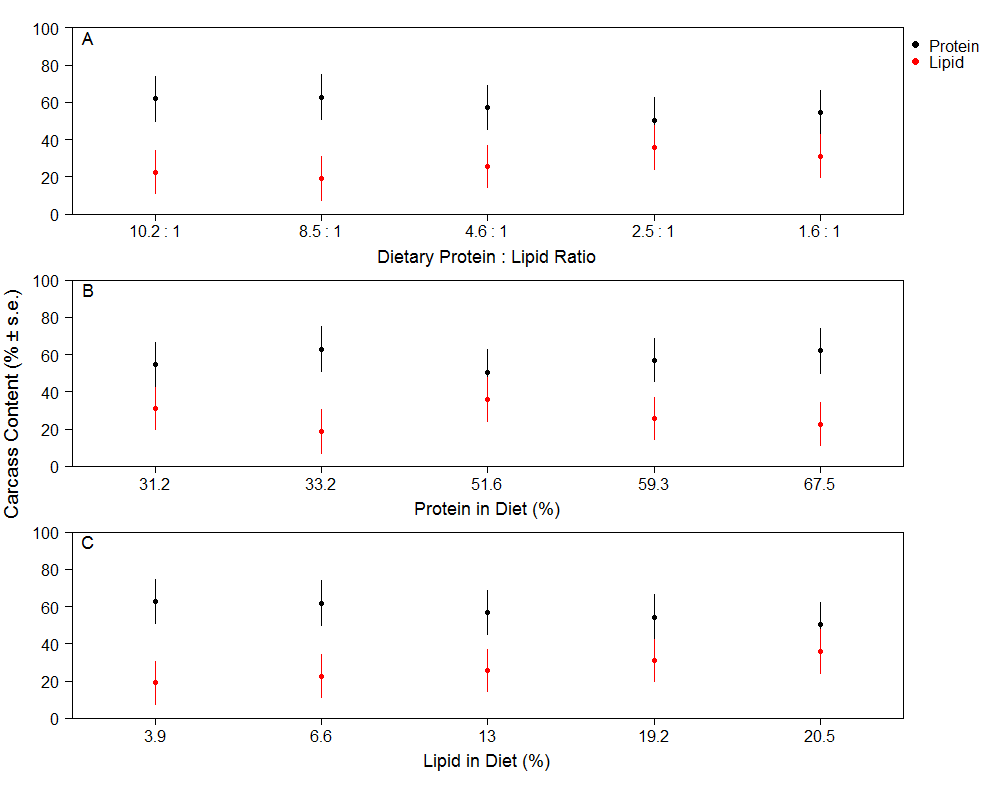
Figure S8.** Mean (% of carcass ± s.e.) carcass protein (black points) and lipid (red points) content in relation to (A) dietary protein : lipid ratio (B) dietary protein content (%) and (C) dietary lipid content (%). Although there is an effect of diet on both carcass protein and lipid content (both *p* < 0.001), this does not follow the rank order of protein to lipid ratios in the diets (panel A). Carcass protein content decreased and carcass lipid content increased with increasing dietary lipid (both *p* << 0.001; panel C). There was no effect of dietary protein on either carcass lipid or carcass protein content (*p*= 0.757 and 0.648 respectively; panel B).
